# Recent Therapeutic Gene Editing Applications to Genetic Disorders

**DOI:** 10.3390/cimb46050255

**Published:** 2024-04-30

**Authors:** Eric Deneault

**Affiliations:** Regulatory Research Division, Centre for Oncology, Radiopharmaceuticals and Research, Biologic and Radiopharmaceutical Drugs Directorate, Health Products and Food Branch, Health Canada, Ottawa, ON K1A 0K9, Canada; eric.deneault@hc-sc.gc.ca

**Keywords:** gene editing, CRISPR, genetic disorders, off-target

## Abstract

Recent years have witnessed unprecedented progress in therapeutic gene editing, revolutionizing the approach to treating genetic disorders. In this comprehensive review, we discuss the progression of milestones leading to the emergence of the clustered regularly interspaced short palindromic repeats (CRISPR)-based technology as a powerful tool for precise and targeted modifications of the human genome. CRISPR-Cas9 nuclease, base editing, and prime editing have taken center stage, demonstrating remarkable precision and efficacy in targeted ex vivo and in vivo genomic modifications. Enhanced delivery systems, including viral vectors and nanoparticles, have further improved the efficiency and safety of therapeutic gene editing, advancing their clinical translatability. The exploration of CRISPR-Cas systems beyond the commonly used Cas9, such as the development of Cas12 and Cas13 variants, has expanded the repertoire of gene editing tools, enabling more intricate modifications and therapeutic interventions. Outstandingly, prime editing represents a significant leap forward, given its unparalleled versatility and minimization of off-target effects. These innovations have paved the way for therapeutic gene editing in a multitude of previously incurable genetic disorders, ranging from monogenic diseases to complex polygenic conditions. This review highlights the latest innovative studies in the field, emphasizing breakthrough technologies in preclinical and clinical trials, and their applications in the realm of precision medicine. However, challenges such as off-target effects and ethical considerations remain, necessitating continued research to refine safety profiles and ethical frameworks.

## 1. Emergence of Targeted Gene Editing

The revelation that an exogenous gene can be inserted into the desoxyribonucleic acid (DNA) sequence of mammalian cells through homologous recombination [1], and that artificial photoendonucleases can break DNA at a specific sequence [2], laid the foundation for the development of gene targeting techniques. However, the potential of gene targeting for therapeutic use became more plausible with the discovery that the rate of on-target integration by homologous recombination was dramatically increased following the introduction of a specific double-strand break (DSB) near the target site [3]. Following this, cleaving DNA as close as possible to the insertion site became critical for successful gene engineering.

### 1.1. Original Gene Editing Tools

Restriction enzymes were discovered more than 50 years ago and became a pioneering tool for gene editing [4]. Restriction enzymes allow precise gene modification since they can break DNA at specific sequences. Their use has paved the way for the development of recombinant DNA technology, allowing the cut and paste of genetic material from one organism to another. However, most restriction endonucleases have their own unique recognition sequence that is not modifiable or programmable, which reduces the likelihood of finding their corresponding sequence near the desired target site.

The search for new strategies to make more customizable DSBs went on for many years until the development of the first generation of engineered nucleases, namely the zinc finger nucleases (ZFNs). These resulted from the fusion of the nonspecific DNA cleavage domain of the type IIS restriction endonuclease FokI from *Flavobacterium okeanokoites*, which is separate from its DNA binding domain, to several zinc finger protein modules, each interacting with a triplet codon of DNA [5]. Soon after, it was demonstrated that FokI must homodimerize for DNA cleavage to occur, where each monomer contains one catalytic center that cleaves one DNA strand, resulting in a DSB [6]. Since the zinc finger modules are programmable for a specific target DNA sequence, this chimeric ZFN technology was successfully used to edit an inherited mutation in *IL2RG*, causing severe combined immune deficiency (SCID) [7]. These results represented a significant improvement in the field of targeted gene editing.

In the following years, transcription activator-like effector nucleases (TALENs) were established as a new type of FokI chimeric engineered nuclease, originating from *Xanthomonas* bacteria, to provide a more flexible tool for gene editing [8]. Indeed, the modular nature of TALEN repeats are less complex to design and assemble compared to the rigid zinc finger motifs used in ZFNs. Moreover, TALENs generally exhibit reduced likelihood of unintended DNA cleavage at off-target sites. Nonetheless, for any new genomic locus to target, both ZFNs and TALENs necessitate the repetition of the whole process of protein module design, cloning, and assembling, which is time-consuming and impractical for widespread application.

### 1.2. The Missing Piece

**Figure 1:**
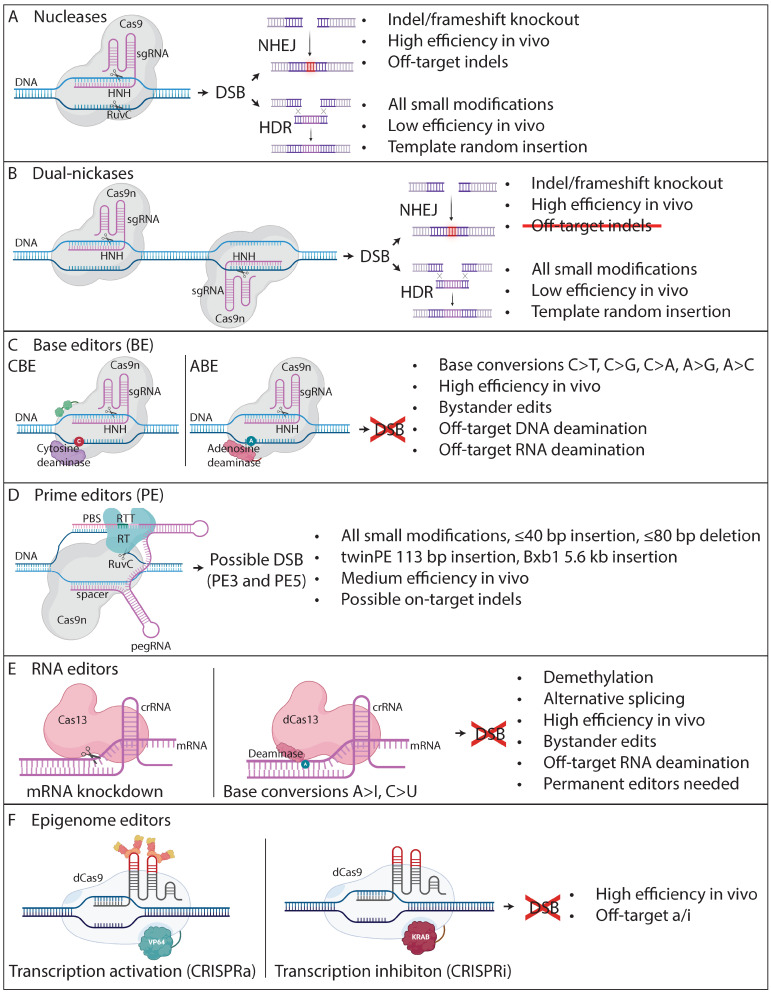
CRISPR-Cas genome editing tools. (**A**) Cas9 nuclease associates with a sgRNA to generate a DSB using its two nuclease domains, i.e., a HNH domain cleaving the complementary strand, and a RuvC-like domain cleaving the non-complementary strand. Then, the DSB is typically repaired via the error-prone NHEJ or error-free HDR pathways, each with described specific characteristics. (**B**) Example of a dual-Cas9n (inactivated RuvC domain) with two sgRNAs creating two nicks in proximity and on opposite strands, resulting in a DSB that is repaired by NHEJ or HDR, without off-target indels. (**C**) CBE, or ABE, involves one Cas9n with inactivated RuvC domain fused to a corresponding deaminase, in addition to a sgRNA, to trigger various types of base conversions, without DSB. (**D**) PE includes a Cas9n with inactivated HNH domain, in combination with a pegRNA, to install different described DNA modifications. (**E**) A crRNA unites with Cas13 to break target mRNA, or with dCas13 fused to a cytosine or adenosine deaminase to stimulate base conversions, without DSB. (**F**) Transcription of target genes can be upregulated with dCas9-VP64, or downregulated with dCas9-KRAB. Cas: clustered regularly interspaced short palindromic repeats (CRISPR) associated protein; dCas: dead Cas; sgRNA: single-guide ribonucleic acid; DNA: desoxyribonucleic acid; HNH: histidine-asparagine-histidine domain; RuvC: RusA endonuclease variant C domain; DBS: double-strand break; NHEJ: non-homologous end joining; HDR: homology-directed repair; indel: small insertion and deletion; CBE: cytosine deaminase; ABE: adenosine deaminase; PBS: primer binding site; RT: reverse transcriptase; RTT: RT template; pegRNA: prime editing guide RNA; bp: base pair; kb: kilobase; CRISPRa: CRISPR activation; CRISPRi: CRISPR interference; crRNA: CRISPR RNA; mRNA: messenger RNA; a/i: activation/interference; A: adenine; T: thymine; G: guanine; C: cytosine; I: inosine.

The breakthrough that finally made targeted gene editing easily programmable and broadly accessible came with the discovery of the CRISPR system with its ribonucleic acid (RNA)-guided endonuclease CRISPR-associated (Cas) protein 9 from the bacteria *Streptococcus pyogenes* [9]. In this CRISPR-Cas9 system, the specificity of DNA targeting is entirely dictated by a short single-guide RNA (sgRNA) molecule with a fully customizable and synthesizable sequence (Figure 1A). Unlike FokI, Cas9 generates a DSB without homodimerization since it contains two nuclease domains, i.e., an histidine–asparagine–histidine (HNH) domain cleaving the complementary strand, and a RusA endonuclease variant C (RuvC)-like domain cleaving the non-complementary strand [9]. Its unprecedented ease of use and widespread effectiveness has been confirmed in several species, including humans [10,11,12]. The simple design and assembly of multiple sgRNAs allowed the creation of genome-wide libraries of sgRNAs, able to target almost any gene for high-throughput screenings [12,13].

## 2. Molecular Mechanisms of CRISPR

The first observation of an unusual structure composed of segments of homologous DNA organized in direct repeats, or clustered repeats, was made in the bacteria *Escherichia coli* and published in 1987 [14]. The significance and functional role of these repeats was not clear at that time and only became apparent in subsequent research, leading to the discovery of the CRISPR system as an adaptive immune system in bacteria and archaea against viruses. The term “CRISPR” appeared for the first time in a scientific publication in 2002 [15].

In fact, the repeated sequences were found to be scattered between non-repetitive DNA sequences, named “spacers”, which are acquired directly from invasive bacteriophages or conjugative plasmids and incorporated into a CRISPR array within the host bacterial DNA [16]. Since a specific spacer-carrier virus fails to infect matching spacer-carrier bacteria, these authors proposed a correlation between CRISPR and adaptive immunity. Some Cas enzymes were shown to capture a small fragment of the invading virus DNA and insert it into the bacterial DNA [17]. The Cas proteins are also responsible for processing the CRISPR array into CRISPR RNA (crRNA), which includes the spacers, during transcription. When a new infection strikes, the corresponding crRNA, also called guide RNA (gRNA), guides a Cas protein to cleave the nucleic acid of the invader at a specific target sequence.

A wide variety of Cas proteins with different functions and biochemical properties have been identified and characterized in recent years in a widespread range of species. Recently, a comprehensive classification system was developed in a wiki format to facilitate the search for Cas enzymes based on their nuclease activities, target conditions, enzymatic properties, and sequence similarity [18]. As mentioned above, Cas9 was the first to be characterized and exploited in human cells [10,11,12].

### 2.1. Harnessing Cas9 for Gene Editing

When the type II CRISPR-Cas9 system was originally described in 2012 [9], the authors presented Cas9 as an unspecific endonuclease guided by a crRNA, which are held together by a trans-activating CRISPR RNA (tracrRNA). The crRNA contains the spacer sequence, typically 20 nucleotides in length, that is complementary to the target DNA sequence. In gene editing, this crRNA sequence is engineered to match the DNA sequence of interest and is merged to the tracrRNA, which together constitute the sgRNA. The simple and inexpensive synthesis of any sgRNA has launched the worldwide use of CRISPR-Cas9 for targeted gene editing in a variety of species.

The target sequence must match the 20 nucleotides at the 5′ end of the sgRNA and be located right next to a protospacer adjacent motif (PAM) composed of the nucleotides NGG. The PAM does not pair with the crRNA, but is necessary for Cas9 to form the RNA–DNA duplex [19]. However, certain mismatches can be tolerated between the crRNA and the target DNA and lead to off-target cleavage in other regions of the genome with high similarity. This may cause unintended damage to other genes via small insertions and deletions (indels) and larger structural variations like translocations, inversions, and large deletions [20].

In addition to Cas9, interesting variations of CRISPR have been discovered and developed as gene editing tools. For example, type V CRISPR-Cas12 (or Cpf1) requires a thymine-rich 5′-TTN PAM, cleaves DNA via a staggered DSB with a 4 or 5-nucleotide 5′ overhang, and does not require tracrRNA [21]. Moreover, type VI CRISPR-Cas13 (or C2c2) is guided by a single crRNA to cleave single-stranded RNA instead of DNA, so it can be programmed to knockdown specific messenger RNAs (mRNAs) [22]. These findings expanded the CRISPR toolkit with additional capabilities for precise genome editing. Considerable efforts are currently being invested to enhance precision, reduce off-target damage, and explore applications of CRISPR in various fields including medicine, agriculture, and biotechnology.

### 2.2. Beyond DSB

The role of CRISPR in gene editing is over as soon as the DNA break is made. The modification of the DNA sequence is taken over by a repair mechanism already present in the host cell. Cas9-induced DSBs are typically repaired by the error-prone non-homologous end joining (NHEJ) pathway, which is active throughout the cell cycle and may result in random indels [10] (Figure 1A). When the size of a resulting indel is not a multiple of three nucleotides, a reading frameshift may be induced in a coding DNA sequence, which can set a premature termination codon (PTC) on the course of the new reading frame. This may produce a truncated transcript that is normally degraded by nonsense-mediated decay (NMD), leading to gene expression “knockout” [23].

Less frequently, DSBs may also be repaired by error-free homology-directed repair (HDR) mechanisms during the late S and G2 phases of the cell cycle, in the presence of a repair template (Figure 1A). While the sister chromatid is normally used as a repair template in non-editing contexts, exogenous DNA like plasmids or synthetic single-stranded oligodeoxynucleotides (ssODNs) can be used for gene editing purposes. For example, HDR has been leveraged frequently in combination with high-fidelity Cas9 nuclease and ssODN to make precise gene modifications and point-mutation corrections in human cells [24,25,26,27,28,29].

## 3. Development of Next-Generation CRISPR-Cas9

Since Cas9 contains two nuclease domains, each cleaving one strand of DNA, point mutations have been introduced in the corresponding catalytic residues, i.e., D10A in RuvC, or H840A in HNH, to convert Cas9 into a DNA nickase (Cas9n) [9,10,12] (Figure 1B). Since Cas9n cuts only one strand, two offset sgRNAs in proximity and on opposite strands are needed to create a DSB, thereby extending the number of required matching nucleotides. As individual nicks are repaired with high fidelity, this approach has significantly reduced the possibility of off-target damage. For example, Cas9n-D10A has been shown to be particularly efficient in minimizing off-target indels in cells lines and mouse zygotes [30]. The efficiency and high fidelity of this dual-nickase system has also been confirmed in various gene modification and knockout experiments [31,32,33,34,35,36].

### 3.1. Base Editing

Base editing (BE) represents an innovative approach in which Cas9n is fused to a deaminase enzyme responsible for the direct conversion of one DNA base pair into another. For example, cytosine base editors (CBEs) trigger C-to-T transition [37], adenine base editors (ABEs) trigger A-to-G transition [38], while others can trigger both simultaneously [39], or even C-to-G, C-to-A, and A-to-C transversions [40,41,42,43,44] (Figure 1C). BE has promptly proven its potential for in vivo correction of different pathogenic mutations associated with various diseases, as described below. By avoiding DSBs, BE prevents the risks of unintended indels. However, while BE is a powerful genome editing tool, it does have certain limitations and challenges. For example, the deaminase enzyme in base editors often has a specific editing window, or range of a few nucleotides, where it can effectively modify the target base. Consequently, it may also edit nearby bases within this window, leading to unintended mutations called “bystander” edits. Despite the fact that BE is designed to be highly specific, it may also induce unintended deamination at off-target DNA sites with high homology. Furthermore, off-target mutations were also detected in cellular mRNA in previous studies using CBE [45,46]. In addition, BE is primarily focused on introducing single nucleotide changes, so it does not allow for the deletion or insertion of larger DNA fragments, which limits its utility for certain types of genomic modifications.

### 3.2. Prime Editing

A remarkable advancement in gene editing is the development of prime editing (PE). PE is a technique that offers high precision and fewer errors compared to Cas9 nuclease or BE, and can make all possible small edits (Figure 1D). It generally does not cause DSBs or on-target indels, with the exception of a few versions of PE. It does not cause off-target indels or bystander mutations. Instead, PE leverages an engineered reverse transcriptase (RT) fused to a Cas9n. This Cas9n-RT complex is guided to the target locus by a prime editing guide RNA (pegRNA) that includes a spacer sequence, which is complementary to the target DNA. The pegRNA also includes a scaffold sequence that is fused to the spacer and merges with Cas9n. The 3′ end of the scaffold is fused to the reverse transcription template (RTT) that includes the edit(s) to be installed by the RT. The 3′ end of the RTT is fused to the primer binding site (PBS) that hybridizes with the 3′ end of the nicked DNA, which serves as a primer for RT to synthesize the new edit(s).

Typically, the length of the RTT and the PBS needs to be optimized for each target. The RTT can be designed to introduce any of the 12 possible base substitutions, as well as ≤40-base pair (bp) insertions and ≤80-bp deletions [47]. As for nuclease and BE, the DNA recognition domain of Cas9 is used for PE, so target sequences need to be adjacent to a PAM, which may represent a challenge if one is not available close to the target DNA locus. PE2 is an improved version of the original PE system using an engineered RT to increase editing efficiency, and PE3 nicks the non-edited strand to further increase efficiency by stimulating the de novo synthesized 3′ flap to be used as a repair template instead of the original 5′ flap [47]. However, PE3 sometimes generates unwanted indels since it may produce DSBs by simultaneously nicking both DNA strands. PE4 and PE5 correspond to PE2 and PE3, respectively, with the additional expression of an engineered DNA mismatch repair (MMR)-inhibiting protein that increases editing efficiency [48]. In this same report, PEmax was shown to improve editing rate in synergy with PE4, PE5, and engineered pegRNAs (epegRNAs). Furthermore, PE6 emerged as shorter than PEmax by 0.5–0.8 kilobase (kb), with a 22-fold increased editing efficiency, following phage-assisted protein evolution experiments [49]. Recently, extensive optimizations have been made to drive more efficient in vivo PE, including the use of chicken beta-actin hybrid intron (Cbh) promoter, a trimmed modified prequeosine1-1 riboswitch aptamer (tevopreQ1) sequence fused on the 3′ end of the pegRNA, codon optimization of RT for improved expression, removal of the RT RNaseH domain, mutations in Cas9 for enhanced nickase activity, and optimized nuclear localization signals (NLS), with all of these resulting in the v3em PE3 system [50].

For larger insertion of DNA sequences, twin prime editing (twinPE) can be applied using a pair of pegRNAs located farther away from one another. TwinPE has demonstrated the ability to allow the insertion of a 113 bp sequence between the two pegRNA-directed nicks [51] (Figure 1D). Moreover, this study has used twinPE in combination with the site-specific serine integrase Bxb1, which triggers directional recombination between the attachment sites attB and attP to insert a 5.6 kb DNA donor plasmid into the genome (Figure 1D). This system was also able to generate the inversion of a 40 kb sequence. In another study, programmable addition via site-specific targeting elements (PASTE) leveraged the fusion of Cas9n to both RT and Bxb1, and was found to promote the integration of a 36 kb sequence into the genome [52]. Tn7-like transposons such as CRISPR-associated transposases (CASTs) have been discovered as effectors of RNA-guided DSB-free DNA integration of multi-kilobase sequences in human cells [53,54]. However, CASTs will benefit from future improvements, given that they have shown limited efficiency (<1%) so far, and they possibly leave scars and off-target insertions behind. Furthermore, multiplex automated genome engineering (MAGE) in eukaryotes involves single-stranded DNA-mediated recombineering, and can incorporate fragments of several kb in length [55].

### 3.3. RNA Editing

RNA bases can also be edited to avoid permanent changes or genomic off-targets. For example, the RNA-targeting type VI Cas13 can associate with a specific crRNA and cleave a target mRNA molecule to knockdown the expression of the coding gene (Figure 1E). Moreover, the fusion of an inactivated form of Cas13 (dCas13) to adenosine deaminases acting on RNA (ADAR) enzymes can deaminate adenosine into inosine (A-to-I) that is recognized as a guanine, and fusion to Cas9-mediated RNA base editing (ceRBE) can induce A-to-I and C-to-U conversions, without RNA cleavage [56] (Figure 1E). However, since mRNA is continuously transcribed from DNA and typically has a short life, the RNA editing machinery must be permanently expressed for a long-term effect, which may increase collateral bystander and genomic off-target edits.

### 3.4. Epigenome Editing

Both D10A and H840A mutations in Cas9 can be simultaneously introduced to create dead Cas9 (dCas9) [9], which is devoid of any endonuclease activity but holds the potential to interfere with target gene transcription by steric hindrance, in a process named CRISPR interference (CRISPRi) [57]. Various effector proteins have been fused to dCas9 to leverage its versatile DNA binding properties and induce changes that do not involve its own endonuclease activity. For example, effector proteins inducing epigenetic modifications have been fused to dCas9 to control the expression levels of target genes without altering the underlying DNA sequence. Epigenome editing involves precise modifications to epigenetic markers, such as DNA methylation and histone modifications, offering potential treatments for diseases influenced by epigenetic factors. For example, the repressive effector Krüppel associated box (KRAB) domain of Kox1 was fused to dCas9 to repress target gene expression by CRISPRi, while the transcription activator VP64 was fused to dCas9 to activate gene expression by CRISPR activation (CRISPRa) [58] (Figure 1F).

So far, the six above-mentioned groups of genome editors, i.e., nucleases, dual-nickases, BE, PE, RNA editors, and epigenome editors have made their way into promising preclinical and clinical trials to make precise therapeutic genome modifications. Gene editing fixes the root cause of various diseases, as described below in the section ‘Applications of Therapeutic Gene Editing’. Among the top three most applied genome editors, nucleases are widely used for frameshift-induced gene knockouts, BE is well suited for point mutation corrections, and PE can address a wide range of mutation classes and sizes.

## 4. Delivery Systems for Gene Editing Tools

A considerable limitation in therapeutic gene editing relates to maintaining the efficacy and safety of delivering CRISPR-based editors into specific target cells without causing unintended damage. Improving the delivery of editing tools to target cells has been an important recent focus of innovation. Novel delivery systems, including viral vectors and nanoparticles, aim to increase the efficiency and specificity of gene editing while minimizing off-target effects.

### 4.1. Viral Vectors

Viral vectors are widely used in therapeutic gene editing as they efficiently deliver genetic material into target cells. Several types of viral vectors have been developed for ex vivo cell-based gene editing, where the editing occurs in cultured cells harvested from the patient before being re-infused, as well as for in vivo gene editing, where the editing tools are delivered directly into the body. The choice of viral vector depends on the specific requirements of the therapeutic application, such as the target cell type, duration of expression, cargo capacity, and safety considerations. Adeno-associated viral (AAV) and lentiviral vectors are the two most widely used vectors in therapeutic gene editing. Both of these vectors have distinct characteristics that make them suitable for various therapeutic applications.

Recombinant AAV vectors are most widely used for in vivo gene editing, given their high transduction efficiency in several cell types, including both dividing and non-dividing cells in various tissues, along with their low immunogenicity and prolonged transgene expression [59]. The capsid determines the tropism, or tissue specificity, for each AAV serotype. Hundreds of AAV serotypes of primate origin have now been described. AAV-based vectors generally carry single-stranded DNA (ssDNA) and can only package ~4.7 kb of transgene cargo, which can complicate all-in-one delivery since the size of the gene encoding Cas9 from *Streptococcus pyogenes* (SpCas9) alone is 4.2 kb. However, one solution to overcome this limitation is using genes encoding other smaller Cas9 variants with comparable efficiencies, such as SaCas9 from *Staphylococcus aureus*, sized at 3.1 kb [60]. Alternatively, it is possible to split the CRISPR machinery using a dual-AAV approach, with very high efficiency for in vivo gene editing in various cell types [50].

In addition, lentiviral vectors are frequently used for both ex vivo and in vivo gene editing since they are capable of integrating into the host genome for stable and long-term transgene expression, transducing both dividing and non-dividing cells, with very low immunogenicity and a larger cargo capacity of ~10 kb [59]. Since the potential for insertional oncogenesis and adverse effects is high with typical lentiviral vectors, a modified version was developed, known as integration defective lentivirus (IDLV), to prevent vector integration for safer in vivo gene delivery [61].

### 4.2. Non-Viral Methods

Non-viral ex vivo gene editing allows the delivery of components through very efficient laboratory methods, such as electroporation and microinjection. However, due to physical constraints, these methods are not applicable to most in vivo gene editing experiments. Instead, various types of nanoparticles, either lipid-, polymer-, peptide-, or inorganic-based, as well as extracellular vesicles (EVs), have been engineered and provide different levels of efficiency for the in vivo delivery of gene editing components. Compared to viral vectors, nanoparticles present a much lower immunogenicity potential, lower manufacturing costs, and more adaptable cargo capacities [59]. For example, lipid nanoparticles (LNPs) are colloidal systems typically less than 100 nm in diameter, made of various types of lipids to encapsulate and protect a nucleic acid payload, facilitating targeted delivery to specific cells. LNPs have gained significant attention in the development of mRNA vaccines, but have also been utilized in many preclinical gene editing therapy studies, showing remarkable efficiency in the delivery of CRISPR-Cas9 systems [62]. LNPs can have varying efficiencies depending on the desired target tissue. For example, they have been shown to be particularly efficient in delivering gene editing payloads to the liver, since hepatocytes highly express low-density lipoprotein (LDL) receptors [63]. New strategies, such as selective organ targeting (SORT) nanoparticles, are being developed to improve tissue-specific targeting efficiency [64]. Even though nanoparticles possibly avoid detection by the immune system better that viral vectors, a significant concern relates to their low selectivity, which may result in the collateral targeting of germline cells in any fertile patient, and pass on the edits to subsequent generations.

## 5. Importance of Therapeutic Gene Editing in Medicine

Most previous clinical trials using gene therapies have involved the viral delivery of a transgene into a patient’s cells to compensate loss-of-function mutations in a defective gene. Such gene replacement therapies are ideal to treat recessive monogenic diseases. However, dominant mutations require corrective gene editing instead of gene replacement. As explained above, next-generation gene editing tools can now achieve targeted deletion, insertion, or correction of gene sequences. Highly precise modifications of DNA or RNA are within reach and can enable targeted corrections of specific genetic mutations associated with various diseases. This level of precision is crucial in developing personalized treatments.

In addition to precise correction of genetic mutations, gene editing technologies can be employed in the development of innovative cancer therapies by arming the patients’ own immune cells with ammunitions specifically tailored to recognize and destroy cancer cells. Editing cancer driver mutations also represents an interesting approach for cancer therapy; however, current CRISPR-Cas9 gene editing tools may not induce sufficient editing rates, leaving behind unedited cancer cells able to rapidly reconstitute the tumor. Gene editing can also disrupt pathogenic viral DNA, or make immune cells resistant to viral replication.

The ultimate goal of gene therapies is to correct the root cause of genetic diseases in single-dose cures, with life-lasting effects and improved quality of life. Gene editing has the potential to reduce the burden on healthcare systems by offering long-term or permanent treatments rather than symptom management.

## 6. Applications of Therapeutic Gene Editing

There has been a global increase in preclinical studies and clinical trials testing gene editing therapies to cure various genetic disorders, cancers, and infectious diseases. A large number of studies have already reported the immense potential of various CRISPR gene editing tools to repair an increasing list of genetic defects related to different diseases. This section highlights examples of promising preclinical therapeutic advances and clinical trials (from clinicaltrials.gov) using ex vivo and in vivo CRISPR-based gene editing to treat genetic disorders, mostly delivered by AAV vectors or LNPs, that have shown significant benefits in humans or in transgenic mouse models carrying a human version of the disease gene. A series of scientific studies are described in regard to disease-relevant mutations, implementation of gene editing systems that target different tissues and organs, editing efficiencies, and the extent of unintended damage. Such damage may involve off-target indels as a result of cleavage of other genomic regions having only a few mismatches with the sgRNA. Undesired damage may also include monoallelic and biallelic on-target indels, and even larger deletions and complex rearrangements. Bystander edits introduced at nearby bases within the editing window of BE are also considered unintended damage, as well as off-target deamination of RNA and DNA.

Rising success is being observed relating to therapeutic gene editing in chimeric antigen receptor in T-cells (CAR-T) therapy, cancer, and infectious diseases. However, these large topics will not be covered in this review.

### 6.1. Blood Diseases

Ex vivo gene editing has proved successful in the treatment of specific blood diseases by targeting the underlying genetic defects responsible for these conditions. In vivo gene editing approaches are also being developed for blood diseases (Table 1).

#### 6.1.1. Sickle Cell Disease and Transfusion-Dependent Beta-Thalassemia

Casgevy™ (exagamglogene autotemcel) was the first CRISPR gene editing therapy in the world to receive regulatory approval for clinical use, granted by the United Kingdom Medicines and Healthcare Products Regulatory Agency in late 2023. Casgevy™ is a cell-based gene therapy developed by Vertex Pharmaceuticals, Boston, USA, and CRISPR Therapeutics, Zug, Switzerland, for the treatment of sickle cell disease (SCD) and transfusion-dependent beta-thalassemia (TDT) [65]. SCD and TDT present deficient hemoglobin activities in red blood cells. Casgevy™ was developed from CTX001, an investigational, autologous ex vivo CRISPR-Cas9 gene editing therapy, in which hematopoietic stem cells (HSCs) from patients with TDT or severe SCD were edited to produce high levels of fetal hemoglobin (HbF) in red blood cells [66]. In this ex vivo gene editing strategy, patient’s CD34+ hematopoietic stem and progenitor cells (HSPCs) were electroporated to introduce SpCas9 nuclease and sgRNA designed to create NHEJ-based indels to disrupt an erythroid-specific enhancer within *BCL11A*. This gene encodes a transcription factor responsible for the repression of gamma-globin and HbF expression in erythroid cells. This approach led to 80% editing (Table 1) and increased HbF production, sufficient to relieve symptoms associated with TDT and SCD, such as vaso-occlusive episodes. A careful analysis of potential off-target damage was performed using in silico and in vivo methods, and there was no evidence of off-target editing. These results were supported by several clinical trials (NCT03655678, NCT03745287, NCT05356195, NCT05329649, NCT05951205, NCT05477563, and NCT04208529).

**Table 1:** List of example gene editing strategies used in preclinical studies and clinical trials for blood diseases.

Disease	Target Gene	Editor	Delivery	Editing Efficiency	Significant Unintended Edits	Drug	Clinical Trials	Sponsor	Reference
Sickle cell disease (SCD) and transfusion-dependent beta-thalassemia (TDT)	*BCL11A*	SpCas9 nuclease	Electroporation in CD34+ human cells, ex vivo	80%	None	CTX001	NCT03655678, NCT03745287, NCT05356195, NCT05329649, NCT05951205, NCT05477563, NCT04208529	Vertex Pharmaceuticals and CRISPR Therapeutics	[66]
SCD/TDT	*HBG1/2*	AsCas12a nuclease	CD34+ human cells, ex vivo	80%	None	EDIT-301	NCT05444894 and NCT04853576	Editas Medicine	n/a
SCD/TDT	*HBG1/2*	ABE8s	Electroporation in CD34+ human cells, ex vivo	60%	None	n/a	n/a	n/a	[67]
SCD/TDT	*HBB*	ABE8e	Electroporation in CD34+ human cells, ex vivo	68%	2% bystander	n/a	n/a	n/a	[68]
SCD/TDT	n/a	ABE	LNPs anti-CD117, in vivo	n/a	n/a	n/a	n/a	n/a	[69]
SCD/TDT	*HBB*	PE3	Microinjection in mouse IVS-II-654 zygotes, ex vivo	14%	None in HEK293T cells	n/a	n/a	n/a	[70]
SCD/TDT	*HBB*	PE5max	HDAd5/35++ in mouse CD46/Townes, in vivo	40%	1.5% on-target indels	n/a	n/a	n/a	[71]
Severe combined immunodeficiency (SCID)	*CD3D*	ABE8e and ABEmax	Electroporation in CD34+ *CD3D*-humanized mouse cells, ex vivo	88%	ABE8e 50% bystander; ABEmax 1.4% bystander	n/a	n/a	n/a	[72]
Chronic Granulomatous Disease (CGD)	*NCF1*	PE	Electroporation in CD34+ human cells, ex vivo	75%	None	n/a	n/a	n/a	[73]
Hemophilia	*Serpinc1*	SpCas9 nuclease	LNP in mouse *F8^I22I^* and *F9^Mut^*, in vivo	22–38%	None	n/a	n/a	n/a	[74]
Hemophilia	*Serpinc1* and *F9*	SpCas9 nuclease	LNP and AAV8 in mouse *F9^Mut^*, in vivo	20% indel; 3% knockin	None	n/a	n/a	n/a	[75]
Hemophilia	*Serpinc1* and *F8*	SpCas9 nuclease	LNP and AAV8 in mouse *F8^I22I^*, in vivo	30% indel; 0.13% knockin	n/a	n/a	n/a	n/a	[76]

In parallel, EDIT-301 is being clinically tested by Editas Medicine, Cambridge, MA, USA, using AsCas12a nuclease to create indels at a BCL11A binding site in the HBG1/2 promoter, with 80% editing (Table 1) in CD34+ cells from patients with SCD, increased HbF production, no off-target editing, and reduced cell sickling (NCT05444894 and NCT04853576). Moreover, an evolved version of ABE with higher activity (ABE8s) was developed with a similar goal, i.e., mutating the gamma-globin promoter in human CD34+ cells to increase HbF expression [67]. Interestingly, these authors achieved 60% editing efficiency and avoided any off-target indels that could have been caused by the original Cas9 nuclease method. Minimal levels of off-target deamination were found in both DNA and RNA.

Another iteration of ABE with high activity (ABE8e) was used to correct a SCD pathogenic mutation in the beta-globin gene *HBB* in patient CD34+ cells transplanted into humanized mice, with 68% efficiency (Table 1), minimal bystander edits, and a few off-target mutations of no anticipated clinical relevance [68]. Interestingly, LNPs were engineered to carry anti-CD117 (KIT) antibodies on their surface, and deliver by intravenous injection an mRNA-encoded ABE system directly to long-term hematopoietic stem cells (LT-HSCs) in vivo, instead of ex vivo, for a near-complete correction of hematopoietic sickle cells (Table 1) [69]. In a different report, PE3 was microinjected into the zygotes of a mouse model for human beta-thalassemia IVS-II-654 mutation (C > T), which resulted in aberrant splicing of the beta-globin gene, with 14% editing efficiency (Table 1), restoration of normal splicing of beta-globin mRNA, and elimination of thalassemia symptoms [70]. These authors identified several mice with off-target damage, which seemed not to occur in human embryonic kidney 293T (HEK293T) cells. Furthermore, a different group performed in vivo prime editing of beta-globin using a nonintegrating HDAd-PE5max vector after HSC mobilization in the SCD mouse model *CD46/Townes*, and obtained 40% editing of beta-globin S alleles in HSCs (Table 1), 43% replaced hemoglobin, reduced SCD phenotypes, 1.5% on-target indels, and no detectable off-target edits [71].

#### 6.1.2. Severe Combined Immunodeficiency

ABE has also demonstrated promising effectiveness in correcting mutations in *CD3D* causing severe combined immunodeficiency (SCID), and restoring normal expression of CD3δ, which is necessary for normal thymopoiesis [72]. In this study, 88% correction was observed in human CD34+ cells from immunodeficient mice (Table 1), and it was shown that CD3/TCR expression could be restored using a 3D artificial thymic organoid system. The authors noted much higher levels of bystander edits produced by ABE8e (50%) compared to the previous version ABEmax (1.4%), and suggested that the latter would be safe for the rescue of healthy T-cell function. Despite finding a few off-target edits in intronic or intergenic DNA regions using in silico and in vitro methods, the authors concluded that these would not be of clinical concern.

#### 6.1.3. Chronic Granulomatous Disease

Chronic granulomatous disease (CGD) is a genetic disorder that affects the ability of certain white blood cells to kill bacteria and fungi, and is caused by mutations in genes responsible for the production of components of the NADPH oxidase enzyme complex [73]. An ex vivo PE strategy was developed to correct an homozygous two nucleotide GT deletion (delGT) in exon 2 of *NCF1*, which encodes the p47phox protein, a subunit of the NADPH oxidase complex [73]. In this study, the autologous CD34+ cells of CGD patients were electroporated with prime editor machinery, which led to 75% correction of at least one allele in CD34+ cells (Table 1) and 80% restoration of p47phox protein expression and NADPH oxidase activity. The authors did not detect any off-target editing or chromosomal rearrangements.

#### 6.1.4. Hemophilia

Hemophilia A and B are characterized by prolonged bleeding episodes, and are caused by deficiencies in the coagulation factors VIII and IX, encoded by *F8* and *F9*, respectively [74]. To treat hemophilia A and B, an LNP-based approach was developed to deliver SpCas9 nuclease mRNA and sgRNA to disrupt the expression of *Serpinc1*, encoding the anticoagulant protein antithrombin (AT), in the liver of hemophilic mouse models *F8^I22I^* and *F9^Mut^* [74]. The authors reported a 22–38% indel frequency rate in the liver (Table 1), as well as a 40–70% reduction in blood AT concentration, leading to improvement in thrombin generation and blood coagulation. No off-target indels were detected at the 10 highest potential sites.

However, since this rebalancing strategy might be insufficient to control acute bleeding, human *F9* knockin (KI), using AAV as a donor for CRISPR-Cas9, was used in combination with the *Serpinc1* knockout method described above [75]. In this study, a hybrid system of LNP-packed sgRNA/SpCas9 mRNA and AAV8-packed *F9* donor template was used in the hemophilia B mouse model, and led to a 20% indel rate at the on-target site (Table 1). Blood AT levels were reduced by 67%, and coagulation was restored to normal levels. Integration of human *F9* occurred in 3% of the target locus in hepatocytes. No indels were detected in the ten off-target candidate sites selected. Furthermore, a very similar hybrid strategy was developed to disrupt *Serpinc1* while knocking in human *F8* in the mouse model for hemophilia A [76]. The authors reported a 30% on-target indel rate and 0.13% KI rate for *F8* (Table 1).

### 6.2. Neurological Disorders

Therapeutic gene editing holds promise in addressing neurological disorders like amyotrophic lateral sclerosis (ALS), Alzheimer’s disease (AD), Huntington’s disease (HD) and Niemann-Pick disease (Table 2). By targeting and editing the genes associated with these conditions, researchers aim to slow down or halt disease progression.

#### 6.2.1. Amyotrophic Lateral Sclerosis

ALS is a fatal adult-onset neurodegenerative disorder characterized by a progressive loss of motor neurons in the brain and spinal cord, leading to progressive muscle atrophy, paralysis and death [77]. Dominant mutations in CuZn superoxide dismutase 1 (*SOD1*) are responsible for 20% of the inherited forms of the disease [78]. A few years ago, a proof-of-concept study examined the *SOD1^G93A^* mouse model of ALS, carrying about 25 tandem repeat copies of the human transgene *hSOD1^G93A^* [79]. Following neonatal systemic administration of an AAV9 vector encoding SaCas9 nuclease and an sgRNA, this method created a frameshift-based reduction in the expression of the transgenes hSOD1^G93A^. Despite a low editing rate of 0.4% (Table 2), the authors noted a three-fold decrease in mutant SOD1, improved survival of motor neurons and motor function, reduced muscle atrophy, and 25% prolonged survival. No significant indels were found at the 12 candidate off-target cleavage sites in treated mice. Of note, high off-target activity may not be expected along with low on-target activity.

Also using *SOD1^G93A^* transgenic mice, a different group used AAV9 for in vivo delivery of SaCas9 nuclease and a different sgRNA via intracerebroventricular (ICV) injection [80]. The authors reported ~1.5% editing (Table 2), a notable delay in motor neuron degeneration, and 54% improved lifespan. Moreover, deep sequencing analysis did not reveal any significant off-target damage in mice cells.

In an additional study using the same *SOD1^G93A^* mouse model, the SaCas9 nuclease approach was replaced with a SpCas9-CBE delivered by a split-intein dual-AAV9 system to introduce a specific nonsense mutation, and to prevent DNA breaks, unintended indels, and chromosomal rearrangements [81]. In this work, 1.2% editing was achieved (Table 2), as well as improved survival, slowed muscle atrophy, and late disease progression. No significant off-target base editing was detected when eight candidate off-target sites in genomic DNA were analyzed. However, off-target RNA deamination was not monitored here, despite previous reports raising this issue with CBE [45,46]. As such, the development of tools to suppress off-target DNA and RNA base editing, while increasing on-target editing, is critical for advancing gene editing therapies to the clinic.

**Table 2:** Gene editing strategies for neurological, ophthalmic and auditory disorders in preclinical testing and clinical trials.

Disease	Target Gene	Editor	Delivery	Editing Efficiency	Significant Unintended Edits	Drug	Clinical Trials	Sponsor	Reference
Amyotrophic lateral sclerosis (ALS)	*SOD1*	SaCas9 nuclease	AAV9 in mouse *SOD1^G93A^*, in vivo	0.4%	None	n/a	n/a	n/a	[79]
ALS	*SOD1*	SaCas9 nuclease	AAV9 in mouse *SOD1^G93A^*, in vivo	~1.5%	None	n/a	n/a	n/a	[80]
ALS	*SOD1*	CBE	Split-intein dual-AAV9 in mouse *SOD1^G93A^*, in vivo	1.2%	None; (Off-target RNA not looked)	n/a	n/a	n/a	[81]
Alzheimer’s disease (AD)	*APP*	SpCas9 nuclease	AAV9 in mouse *APPswe*, in vivo	2%	n/a	n/a	n/a	n/a	[82]
AD	*Bace1*	Cas9 nuclease	Amphiphilic nanocomplex in mouse 5XFAD, in vivo	45%	None	n/a	n/a	n/a	[83]
AD	*MAPT*	NG-ABE8e	Trans-splicing AAV9 in mouse PS19, in vivo	5.7%	0.35% bystander	n/a	n/a	n/a	[84]
AD	*APOE3*	v3em PE3	Split-intein dual-AAV9 in mouse *APOE3*, in vivo	14%	5% on-target indels	n/a	n/a	n/a	[50]
Huntington’s disease (HD)	*HTT*	SpCas9 nuclease	Dual-AAV2 in mouse BacHD, in vivo	n/a	None	n/a	n/a	n/a	[85]
HD	*HTT*	Cas9 nuclease	Dual-AAV in mouse HD140Q-KI, in vivo	n/a	None	n/a	n/a	n/a	[86]
HD	*HTT*	SaCas9 nuclease	AAV1 in mouse R6/2, in vivo	6%	None	n/a	n/a	n/a	[87]
HD	*HTT*	dCas9-KRAB	Lentivirus in mouse R6/2, in vivo	n/a	None	n/a	n/a	n/a	[88]
Niemann-Pick disease type C (NPC)	*Npc1*	CBE	Split-intein dual-AAV9 in mouse *Npc1*^I1061T^, in vivo	48%	None; (Off-target RNA not looked)	n/a	n/a	n/a	[89]
Leber congenital amaurosis (LCA) 2	*Rpe65*	NG-ABE	Split dual-AAV2 in mouse rd12, in vivo	82%	21% bystander	n/a	n/a	n/a	[90]
LCA2	*Rpe65*	SpCas9-NG nuclease (HDR)	Trans-splicing AAV2 in mouse rd12, in vivo	1%	17% indels	n/a	n/a	n/a	[91]
LCA2	*Rpe65*	SpCas9-NG-PE2	Trans-splicing AAV2 in mouse rd12, in vivo	28%	None	n/a	n/a	n/a	[91]
LCA2	*Rpe65*	PE3	Split dual-AAV8 in mouse rd12, in vivo	16%	None	n/a	n/a	n/a	[92]
LCA10	*CEP290*	SaCas9 nuclease	AAV5 in mouse and monkey *CEP290*, in vivo	Above therapeutic threshold	None	EDIT-101	NCT03872479	Editas Medicine	[93]
Retinitis pigmentosa (RP)	*Pde6b*	PE-SpRY	Split Npu intein dual-AAV in mouse *Pde6b^rd10^*, in vivo	76% in transduced cells	None	n/a	n/a	n/a	[94]
RP	*Rho*	Cas12f1 nuclease	AAV in mouse *Rho^P23H^*, in vivo	70% in transduced cells	Minimal bystander	ZVS203e	NCT05805007	Peking University Third Hospital	[95]
Congenital hearing loss	*OTOF*	dCas13X	AAV9 in mouse *OTOF^Q829X^*, in vivo	80%	None	HG205	NCT06025032	HuidaGene Therapeutics	[96]
Congenital hearing loss	*Htra2*	CasRx	AAV-PHP.eB in mouse model, in vivo	82% knockdown	Low	n/a	n/a	n/a	[97]
Congenital hearing loss	*Myo6*	dCas13X.1-ABE (mxABE)	AAV-PHP.eB in mouse *Myo6^C442Y/+^*, in vivo	4%	None	n/a	n/a	n/a	[98]

#### 6.2.2. Alzheimer’s Disease

The KM670/671NL (APPswe [Swedish]) dominant mutation is located at the β-secretase (BACE1) cleaving site in the amyloid beta precursor protein (APP) and causes increased cleavage of the amyloid-β (Aβ) precursor protein, potentially leading to inherited AD [99]. In a preclinical study, SpCas9 nuclease and a mutant allele-specific sgRNA were delivered using two separate AAV9 vectors to create indels, and to knockout the expression of the *APPswe* allele after co-injection into hippocampus of adult *APPswe* transgenic mice [82]. These mice carry approximately 100 copies of the human transgene *APPswe*, in which the authors detected about 2% editing efficiency within the injected area (Table 2). The results of this experiment revealed reduced levels of pathogenic Aβ secretion. Since the pathology evolves gradually over a long period of time, even a small fraction of allelic disruption could be therapeutically beneficial. This approach may have the potential to develop as a gene therapy against *APPswe*-derived AD. However, potential off-target indels generated by Cas9 nuclease were not scrutinized in this study, which is necessary before moving ahead to clinical studies.

In a different study, Cas9 nuclease and sgRNA loaded into an amphiphilic nanocomplex were injected into the hippocampus of the mouse model 5XFAD to disrupt *Bace1* in post-mitotic neurons [83]. They obtained a 45% editing rate (Table 2), a significant reduction in Aβ plaque accumulation, and no detected off-target indels.

A BE strategy was used to correct the pathogenic mutation P301S in the microtubule-binding protein tau, encoded by *MAPT*, in the PS19 transgenic mouse model for AD [84]. These authors used trans-splicing AAV9 to deliver NG-ABE8e, which is a fusion of SpCas9-NG and an evolved TadA monomer, in combination with a sgRNA, in the hippocampi of PS19 mice. They obtained the expected A-to-G substitution with 5.7% efficiency (Table 2), with 0.35% bystander edits. A significant reduction in pathogenic tau levels was observed, as well as improved cognitive function. No evidence of off-target effects was found. An interesting PE approach (i.e., the split-intein v3em PE3-AAV9 system) was used by another group to install the putatively AD-protective apolipoprotein E Christchurch (*APOE3*) R136S variant, a G-to-T transversion mutation that cannot be installed using BE, or with low efficiency using HDR in post-mitotic cells, into humanized *APOE3* mice by ICV injection [50]. This strategy led to 12% precise editing efficiency with 5.0% on-target indels in the bulk neocortex, and 14% editing with 3.1% indels in hippocampi for P1 injected mice (Table 2). No off-target indels were detected. These results indicate modest but promising in vivo editing potential for this protective variant; however, the presence of on-target indels represents an obstacle for a safe gene editing therapy.

#### 6.2.3. Huntington’s Disease

Huntington’s disease (HD) is a neurodegenerative disorder caused by CAG trinucleotide tandem repeats encoding a polyglutamine (polyQ) tract in the N-terminal region of the huntingtin gene (*HTT*) [100]. A research group developed an allele-specific gene editing approach using AAV2 vectors to deliver SpCas9 nuclease and a pair of sgRNAs to create small-targeted deletions to knockout HTT expression in the BacHD mouse model, which is transgenic for human mutant huntingtin (mHTT), containing 97 CAG repeats (Table 2) [85]. The authors showed that their strategy could specifically reduce the production of toxic mHTT proteins to 40%, i.e., without affecting the wild-type allele. However, this strategy implies that a relevant PAM-disrupting mutation would have to be identified in each patient, which may not always be straightforward. No indels were found at the 11 top candidate off-target cleavage sites in human HD fibroblasts.

A distinct preclinical study took advantage of the mouse model HD140Q-KI, in which exon 1 of *Htt* was replaced with exon 1 of human *HTT* with 140 CAG repeats [86]. The authors used a dual-AAV approach to deliver Cas9 nuclease and a few non-allele-specific sgRNAs using stereotaxic injection into the mouse striatum to generate a frameshift-induced knockout (Table 2). They found reduced mHTT levels, improved motor functions, and permanent elimination of polyQ expansion-mediated neuronal toxicity in the adult brain. However, whether reducing the expression of endogenous *HTT* can be used to treat HD patients without deleterious effects remains unknown. Their results also suggest that mature neuronal cells are able to clear accumulated mHTT and repair related injury. Thus, reducing mHTT expression in the brain of elder HD patients could moderate neurological symptoms. In addition, whole-genome sequencing was used to confirm the lack of off-target indels in the injected mouse striatum.

Another group later published the results of a study in which a different *HTT* mouse model (R6/2, carrying exon 1 of human *HTT* with 115–150 CAG repeats) was used, as well as SaCas9 packaged with non-allele-specific sgRNA in a single AAV1 vector to create indels and knockout the expression of *mHTT* [87]. The authors observed 6% editing in vivo (Table 2), a 30% reduction in mHTT levels, as well as significant improvements in lifespan and motor deficits. No indels were found at the 10 candidate off-target cleavage sites in mice.

Alternatively, a double-strand break (DBS)-free CRISPRi approach was designed to silence *mHTT* in R6/2 mice to avoid any DNA damage and off-target indels [88]. In this study, since KRAB-associated repressive histone marks are labile, a permanently active lentiviral vector was used for continuous expression of dCas9-KRAB (Table 2). The authors reported that CRISPRi targeting the CAG repeat region significantly decreased mHTT expression while preserving wild-type HTT expression. HD progression was delayed, according to several behavioral tests. Off-target activity at susceptible sites was not detected. CRISPRi may represent an interesting therapy for HD that can avoid recurring issues associated with Cas9 nuclease, such as on- and off-target indels.

#### 6.2.4. Niemann-Pick Disease

In a mouse model of human Niemann-Pick disease type C (NPC), which is a neurodegenerative lipid storage disorder, the responsible genetic mutation *Npc1*^I1061T^ was targeted for correction using CBE [89]. After neonatal administration of an optimized split-intein dual-AAV9 CBE, the authors monitored a modest but significant lifespan extension, with up to 48% editing in cortical cells (Table 2). No bystander C-to-T edits were found among edited alleles, and over 94% of the edited alleles precisely corrected the I1061T mutation. Looking at eight candidate off-target sites in mice, they confirmed a single off-target mutation in an intronic sequence, more than 3 kb away from the nearest exon. However, off-target RNA deamination was not evaluated with their CBE system, which is important for further clinical studies.

### 6.3. Ophthalmic Disorders

Gene editing offers potential treatments for inherited eye disorders, such as Leber congenital amaurosis (LCA), retinitis pigmentosa (RP), and certain forms of inherited blindness. Correcting genetic mutations in retinal cells could restore vision. Retinal diseases are appealing targets for gene therapies since the anatomical structure is easily accessible and less subject to an immune response. To this end, numerous preclinical studies and clinical trials have been conducted (Table 2). Below are examples of different gene editing approaches applied in ophthalmic disorders.

#### 6.3.1. Leber Congenital Amaurosis

Luxturna^®^ is an approved gene therapy for LCA, an inherited retinal degeneration that causes severe visual dysfunction, using AAV2-hRPE65v2 to deliver a full-sized functional copy of the *RPE65* transgene to patient retinal pigment epithelium (RPE) cells. However, since several patients show progressive retinal re-degeneration in the years following the gene therapy, possibly due to progressive exhaustion of expression of the transgene, a research team used a split enhanced ABE (NG-ABE) dual-AAV2 strategy for in vivo correction of a nonsense mutation in exon 3 of *Rpe65* in the mouse model rd12 for LCA2 [90]. They found rescue of the function and survival of cone photoreceptors on a long-term basis, with 82% A-to-G conversion at on-target adenine (Table 2). However, a 21% rate of bystander editing was observed. The top ten candidate off-target sites did not reveal any off-target editing. Interestingly, also in rd12 mice, a trans-splicing AAV2-SpCas9-NG-PE2 (with wider PAM compatibility) approach has been shown to efficiently correct a point mutation in *Rpe65* with 28% efficiency (Table 2), as well as improving visual functions, without any detectable off-target damage, while the use of a SpCas9-NG nuclease and HDR approach has led to 1% editing and 17% indels (Table 2). Another group used dual-AAV8 split-PE3 to correct *Rpe65* in rd12 mice with 16% efficiency (Table 2), restoration of Rpe65 expression, and rescue of visual function, without detectable off-target edits [92].

EDIT-101, developed by Editas Medicine, is an investigational gene editing therapy administered via subretinal injection in participants with LCA10 caused by a homozygous or compound heterozygous mutation involving c.2991+1655A>G in intron 26 of *CEP290*. SaCas9 nuclease and two sgRNAs specific to human *CEP290* were packaged into AAV5 for subretinal delivery in humanized *CEP290* mice, and in a surrogate non-human primate. The treatments were able to remove an aberrant splice donor created by the mutation, and restore normal CEP290 expression without any detected damage at candidate off-target sites, supporting clinical trial NCT03872479 (Table 2) [93].

#### 6.3.2. Retinitis Pigmentosa

In the RP mouse model *Pde6b^rd10^*, which is characterized by progressing retinal degeneration, PE-SpRY, with an unconstrained PAM requirement, was delivered into the neural retinas using a split Npu intein-based dual-AAV system [94]. The authors observed 76% editing in transduced retinal cells (Table 2), preservation of photoreceptors, and restoration of PDE6 phosphodiesterase activity, along with rescue of visual function. No significant off-target effects were detected in the mouse retinas.

In the RP mouse model *Rho^P23H^*, an engineered miniature Cas12f1 nuclease system was used to overcome the cargo size limits of AAVs and disrupt the mutant allele. The Cas12f1/sgRNA combination CasMINI_v3.1/ge4.1 was able to fit into a single AAV vector, and was injected subretinally before achieving over 70% editing efficiency in transduced retinal cells in vivo (Table 2) [95]. The results of this study included the rescue of visual function and structural preservation, with minimal off-target edits. In connection with these observations, clinical trial NCT05805007, conducted by Peking University Third Hospital, China, is ongoing for the gene editing drug ZVS203e.

### 6.4. Auditory Disorders

A major form of congenital hearing loss is caused by genetic mutations in *OTOF*, coding for otoferlin, which is important for synaptic vesicle exocytosis in inner hair cells of the cochlea and transmission of auditory signals to the brain [96]. To edit the mutation Q829X, a dCas13X RNA base editor (emxABE) was developed and delivered using AAV9 in humanized *OTOF^Q829X^* mice [96]. This approach led to ~80% editing efficiency (Table 2), near complete restoration of OTOF expression in inner hair cells, and significant improvements in long-term auditory function, without major off-target effects. This strategy has recently been authorized for the clinical trial of HG205, with the identifier NCT06025032, by HuidaGene Therapeutics, Clinton, NJ, USA.

The RNA-targeting type VI CasRx was delivered using AAV-PHP.eB by injection into the inner ears in mouse models of sensorineural hearing loss induced by aminoglycoside to knockdown *Htra2*, which encodes a mitochondrial serine proteinase involved in cell apoptosis [97]. The authors observed 82% knockdown of Htra2 transcripts in cochleae (Table 2), inhibition of apoptosis, less cochlear hair cell loss, and improved auditory function, with low off-target and adverse side effects.

Another RNA-targeting system, a mini dCas13X.1-ABE (mxABE), was delivered by AAV-PHP.eB in the cochlea of the mouse model *Myo6^C442Y/+^*, which recapitulates human phenotypes for dominant-inherited deafness [98]. In this study, 4% RNA correction was achieved (Table 2), along with a decrease in hair cell loss and increase in auditory function, without significant bystander edits or off-target damage.

### 6.5. Muscular Disorders

CRISPR gene editing therapy has demonstrated potential for addressing various genetic disorders affecting muscles (Table 3). Muscular disorders often involve specific genetic mutations in skeletal and cardiac muscles that lead to muscle degeneration, weakness, or other related issues.

#### 6.5.1. Duchenne Muscular Dystrophy

Duchenne muscular dystrophy (DMD) is a fatal X-linked recessive muscle disease caused by mutations in *DMD*, which encodes the dystrophin protein [101]. Early proof-of-principle preclinical studies have used Cas9 nuclease and sgRNA to correct or remove mutant exon 23 and re-frame DMD in the mdx mouse model. For example, Cas9 nuclease HDR-mediated correction with ssODN template, while relatively ineffective in postmitotic cells like myofibers and cardiomyocytes, was quite efficient when CRISPR machinery was injected into germ line cells and generated mosaic animals [101]. Indeed, the authors could reach 100% correction of the *Dmd* gene (Table 3), partial recovery of functional dystrophin protein, and low off-target activity. To address in vivo editing in adult mouse muscle tissue, NHEJ-mediated removal of exon 23 was attempted with SaCas9 nuclease and two sgRNAs (no ssODN here) delivered via tibialis anterior muscle injection of AAV8. This led to 2% precise exon 23 deletion (Table 3), 8% recovery of normal dystrophin, 3% on-target indels within flanking introns, and 1% off-target indels at candidate sites [102]. Of note, previous studies had shown that 4% restoration of dystrophin expression is sufficient to significantly improve muscular function [103]. Two very similar approaches, using AAV9 instead of AAV8, obtained comparable results with SaCas9 nuclease [104], or SpCas9 nuclease [105], with up to 39% editing for SaCas9 (Table 3). Furthermore, in the dog model of DMD deltaE50-MD, SpCas9 was delivered with a sgRNA via AAV9 to target a region adjacent to the exon 51 splice acceptor site, and to correct dystrophin expression by skipping of exon 51 [106]. In these dogs, a 10% indel rate was observed (Table 3), restoration of dystrophin expression reached ~80% of wild-type levels in some muscles after 8 weeks, and no off-target damage was detected.

A split ABE with a dual trans-splicing AAV system was delivered into the skeletal muscles of a mouse model of DMD to correct a nonsense mutation in *Dmd* [107]. The authors reported a precise conversion of the premature stop codon to the original glutamine codon with 3% efficiency (Table 3). Dystrophin expression was restored in 17% of myofibers. Since most myofibers contain multiple nuclei, a high proportion of myofibers can be rescued by a low editing efficiency. No bystander or off-target edits were detectable.

**Table 3:** List of gene editing approaches used in preclinical studies on muscular disorders.

Disease	Target Gene	Editor	Delivery	Editing Efficiency	Significant Unintended Edits	Drug	Clinical Trials	Sponsor	Reference
Duchenne muscular dystrophy (DMD)	*Dmd*	Cas9 nuclease (HDR)	Injection into mouse mdx germ line cells, ex vivo	2–100%	Low	n/a	n/a	n/a	[101]
DMD	*Dmd*	SaCas9 nuclease	AAV8 in mouse mdx, in vivo	2%	3% on- and 1% off-target indels	n/a	n/a	n/a	[102]
DMD	*Dmd*	SaCas9 nuclease	AAV9	39%	Minimal	n/a	n/a	n/a	[104]
DMD	*Dmd*	SpCas9 nuclease	AAV9	n/a	None	n/a	n/a	n/a	[105]
DMD	*Dmd*	SpCas9 nuclease	AAV9 in dog deltaE50-MD, in vivo	10%	None	n/a	n/a	n/a	[106]
DMD	*Dmd*	ABE	Split dual trans-splicing AAV in mouse *Dmd*, in vivo	3%	None	n/a	n/a	n/a	[107]
DMD	*Dmd*	SaCas9 nuclease	myoAAV 1A in mouse mdx, in vivo	25%	n/a	n/a	n/a	n/a	[108]
DMD	*Dmd*	ABEmax	Split-intein dual-AAV9 in mouse ∆Ex51, in vivo	35%	11% bystander	n/a	n/a	n/a	[109]
DMD	*Dmd*	PE3	Nucleofection in iPSCs, ex vivo	54%	n/a	n/a	n/a	n/a	[109]
DMD	*Dmd*	twinPE	Transfection in HEK293 cells, ex vivo	28%	5% on-target indels	n/a	n/a	n/a	[51]
DMD	*Dmd*	adRNAs	Dual-adRNA-AAV8 in mouse mdx, in vivo	3.6%	High off-target edits in HEK293 cells	n/a	n/a	n/a	[110]
DMD	*DMD*	dCas13X.1-ABE (mxABE)	AAV9 in mouse *DMD^E30mut^*, in vivo	84%	Constant bystander edits	n/a	n/a	n/a	[111]
Spinal muscular atrophy (SMA)	*SMN1/2*	ABE8e	Split-intein dual-AAV9 in mouse Δ*7SMA*, in vivo	87%	Low indels and bystander	n/a	n/a	n/a	[112]
SMA	*SMN1/2*	PE3	Nucleofection in iPSCs, ex vivo	29%	None	n/a	n/a	n/a	[113]
Cardiomyopathy	*RBM20*	VRQR-SpCas9-ABEmax	Nucleofection in iPSCs, ex vivo	92%	None	n/a	n/a	n/a	[114]
Cardiomyopathy	*RBM20*	PE3b	Nucleofection in iPSCs, ex vivo	40%	None	n/a	n/a	n/a	[114]
Cardiomyopathy	*Rbm20*	VRQR-SpCas9-ABEmax-	AAV9 in mouse *Rbm20^R636Q^*, in vivo	66%	Low bystander and off-target DNA; no off-target RNA	n/a	n/a	n/a	[114]
Cardiomyopathy	*PLN*	SaCas9 nuclease	AAV9 in mouse *hPLN*-R14del, in vivo	Reduction from 51% to 39% in mutant alleles	Low	n/a	n/a	n/a	[115]
Cardiomyopathy	*Myh6*	ABE8e	Split-intein dual-AAV9 in mouse 129SvEv, in vivo	70%	3.4% bystander in DNA, 5% bystander in RNA, no off-target RNA	n/a	n/a	n/a	[116]
Cardiomyopathy	*Myh6*	VRQR-SpCas9-ABEmax-	Split-intein dual-AAV9 in mouse *Myh6^h403/h403^*, in vivo	35%	None	n/a	n/a	n/a	[117]
RYR1 myopathies	*RYR1*	PE3	Electroporation in myoblasts, ex vivo	59%	n/a	n/a	n/a	n/a	[118]

In 2020, a library of AAV capsid variants with barcoded packaged genomes was screened using next-generation sequencing, leading to the identification of a new AAV9 mutant called AAVMYO that showed higher efficiency and specificity in targeting skeletal muscle, the heart, and the diaphragm following peripheral delivery in mice [119]. Shortly after, another group reported a different screening method, and identified new AAV capsid variants with high muscle potency, namely myoAAV 1A and 2A, across mice, non-human primates, and humans [108]. In the latter report, myoAAVs were used to deliver SaCas9 nuclease and sgRNAs in an mdx mouse model for DMD, to excise exon 23 and re-frame the *Dmd* transcript. The MyoAAV 1A variant was at least 10 times more efficient for systemic muscle transduction compared to AAV9, with 25% editing efficiency (Table 3). Moreover, the myoAAV 2A muscle potency was 128 times higher than AAV9, while maintaining low liver transduction without any evidence of liver toxicity or other adverse effects. The muscular system can now be targeted by AAVs with very high specificity.

Another research team took advantage of a different mouse model of DMD, involving the deletion of exon 51 (∆Ex51 mice), using a split-intein dual-AAV9 approach to deliver ABEmax by intramuscular injection, since ABEs produce less off-target editing than CBEs [109]. In this study, the splice donor site of exon 50 was destroyed and dystrophin was correctly reframed. They noted 35% editing in the mutant mouse muscle genomic DNA, with 11% bystander editing (Table 3). Nonetheless, bystander edits in this particular case occurred in the intron or in the removed exon, and therefore should not affect the corrected dystrophin. Nevertheless, it is important to keep in mind that the removal of a splice site may activate noncanonical splice sites in the area and initiate unexpected effects [120]. No off-target damage was revealed at any of the eight tested candidate off-target sites in the ∆Ex51 mice [109]. Moreover, the expression of dystrophin was restored in 96% of myofibers in the treated muscles. Interestingly, this same study also reported a PE3 strategy to insert 2 bp to restore the *DMD* reading frame by nucleofection into human induced pluripotent stem cells (iPSC)-derived cardiomyocytes, with 54% editing efficiency (Table 3), 40% correct reframing of dystrophin, and arrhythmic calcium traces comparable to those of the healthy control cardiomyocytes [109]. In addition, the twinPE approach was efficiently used to excise a 780-bp sequence containing *DMD* exon 51 in HEK293 cells with 28% editing efficiency (Table 3) and 5% on-target indels, without any detectable off-target damage [51]. This may be an interesting candidate for development into in vivo editing therapies.

The RNA base editing system ADAR, with associated guide RNAs (adRNAs), was delivered in the mdx mouse model for *DMD*, via dual-adRNA-AAV8 injection into the tibialis anterior or gastrocnemius, to edit an ochre stop codon in exon 23 of the dystrophin transcript [110]. This resulted in RNA editing efficiency (TAA>TGG/TAG/TGA) of 3.6% (Table 3), and TAA>TGG of 2.4% in treated mice, with limited 1–2.5% dystrophin protein restoration. Unfortunately, high levels of off-target A>G edits were revealed in HEK293 cells, as well as a general negative impact on mouse health, all of which were likely due to the hyper-active version of adRNAs present. To develop more effective approaches, another team delivered a mini-dCas13X.1-mediated RNA adenine base editing (mxABE) by AAV9 to edit a nonsense point mutation (c.4174C>T, p.Gln1392*) in the genetically humanized mouse model *DMD^E30mut^* [111]. Interestingly, these authors obtained 84% A-to-G RNA editing in vivo (Table 3), converting the mutant UAG stop codons to UGG in DMD mRNA transcripts, in addition to 6–54% rescue of dystrophin expression in the diaphragm, tibialis anterior, and heart muscle. However, even though no significant off-target damage was found, bystander A-to-G edits close to the disease-causing mutation were consistently detected.

#### 6.5.2. Spinal Muscular Atrophy

Spinal muscular atrophy (SMA) is a genetic disorder causing progressive degeneration of motor neurons in the spinal cord and muscle weakness, and is a consequence of mutations in the survival motor neuron 1 (*SMN1*) gene [112]. Similarly to Luxturna^®^ presented above, Zolgensma^®^ is an approved AAV-mediated gene complementation therapy, delivering a full-length SMN cDNA, for which long-term expression persistence is yet to be confirmed. Since several mutations in exon 7 strongly regulate splicing of *SMN2*, which is highly similar to *SMN1*, and lead to increased levels of SMN protein, a team developed a series of different ABE8e strategies using split-intein dual-AAV9 to create these mutations and restore SMN protein levels in the mouse model Δ*7SMA* [112]. In this study, up to 87% conversion of the mutation C6T was achieved using ABE8e (Table 3), which also transformed native *SMN2* to *SMN1*, restored SMN protein levels, rescued motor phenotypes, and increased lifespan. They found low levels of indels and bystander edits, and no significant off-target edits were detected in mouse or human cells. In addition, a PE3 approach was designed to delete the intronic splicing silencer-N1 (ISS-N1) in *SMN2* and rescue full-length SMN expression by nucleofection in a patient-specific iPSC model of SMA [113]. They obtained 29% editing efficiency without any off-target damage (Table 3).

#### 6.5.3. Cardiomyopathy

Dilated cardiomyopathy (DCM) is a myocardial disease, characterized by cardiac enlargement and systolic dysfunction, that can lead to sudden cardiac death [114]. Both ABE and PE were used to efficiently correct the DCM-risk dominant mutations R634Q and R636S in *RBM20* in human iPSCs, as well as the mutation R636Q in the mouse model *Rbm20^R636Q^*, which recapitulates human DCM phenotypes [114]. These authors reported that ABEmax fused to VRQR-SpCas9, which targets NGA PAMs, showed 92% editing efficiency in iPSCs without any significant bystander mutations (Table 3). Also, 40% editing efficiency was achieved in iPSCs using the PE3b system (Table 3), which includes a sgRNA designed to bind only the edited allele, without any off-target damage. Appropriate RBM20 functions were restored in both systems. Moreover, intraperitoneal injection of AAV9 was used to deliver ABEmax-VRQR-SpCas9 in *Rbm20^R636Q^* mice under the control of a cardiac-specific cTnT promoter, in which 66% of RBM20 cDNA transcripts were corrected in cardiomyocytes (Table 3). These rescued mice presented restored RBM20 localization and function, improved cardiac function, as well as an extended lifespan. A few bystander mutations, low off-target DNA editing, and no off-target RNA editing were observed in corrected *Rbm20^R636Q^* mouse hearts.

Mutations in the human phospholamban (*PLN*) gene are linked to familial forms of cardiomyopathy, including arrhythmias, ventricular dilation, and possible sudden cardiac death [115]. The transgenic mice *hPLN-R14del*, carrying one of these mutations (R14del), were treated with AAV9 to deliver SaCas9 nuclease and a sgRNA to specifically create on-target indels and knockout the mutant human allele [115]. The results of this study showed modest editing efficiencies, e.g., reduction from 51% to 39% in mutant alleles in CRISPR-treated *hPLN-R14del* mice, while leaving the wild-type allele intact (Table 3). Treated mice also presented reduced mutant PLN transcript levels and PLN protein expression, partially improved cardiac function, and reduced arrhythmia vulnerability. One potential off-target mutation was found in a non-coding region of the mouse genome.

Hypertrophic cardiomyopathy (HCM) is a genetic disorder characterized by hypertrophy of the heart muscle, often caused by mutations in genes that encode proteins of the sarcomere. It leads to various cardiac complications, including arrhythmias, heart failure, and sudden cardiac death [116]. In the mouse model 129SvEv, carrying one R403Q mutation in *Myh6*, split-intein dual-AAV9 ABE8e was used with the *Tnnt2* promoter for cardiomyocyte-exclusive expression to correct the mutation with over 70% efficiency (Table 3) [116]. This study also related that the edited mice maintained normal cardiac structure and function, as well as reduced cardiac fibrosis. They found 3.4% bystander edits in DNA and 5% in RNA in cardiac tissues, as well as off-target edits in intergenic or intronic regions. In non-cardiac tissues, low levels of on-target editing were observed, with insignificant bystander edits. No off-target RNA editing was observed.

In a similar report, VRQR-ABEmax was used in a *Myh6^h403/h403^* mouse model to correct the mutation R403Q [117]. This treatment was shown to be sufficient to prevent the onset of HCM phenotypes. Cardiac tissue showed 35% editing (Table 3), and low levels of editing were observed in non-cardiac tissue. Moreover, no bystander editing, and no significant off-target DNA or RNA editing, was detected in these ABEmax-treated mice.

### 6.6. Premature Aging Diseases

The Hutchinson–Gilford progeria syndrome (HGPS or progeria) is caused by a mis-splicing dominant-negative mutation (G608G; c.1824 C>T) in *LMNA*, generating toxic progerin, which is responsible for accelerated aging and limits the lifespan of children to approximately 14 years [121]. A study described a gene editing therapy in transgenic mice, homozygous for the human *LMNA* c.1824 C>T allele, that recapitulates HGPS symptoms, using retro-orbital injection of split-intein dual-AAV9 to deliver ABEmax at postnatal day 14 [122]. They observed 30% editing in bulk heart and skeletal muscle, 20% in aorta and bone, 55% in bulk liver (Table 4), as well as restored normal RNA splicing, reduced progerin protein levels, and extended lifespan. No off-target DNA or RNA base editing was found, but around 15% bystander editing was found in heart tissues.

### 6.7. Metabolic Diseases

Metabolic diseases, also known as inborn errors of metabolism, encompass a wide range of genetic disorders that affect the body’s normal metabolic processes (Table 4). These disorders typically result from defects in enzymes or other proteins involved in metabolic pathways, leading to abnormalities in the processing of various substrates.

#### 6.7.1. Hypercholesterolemia

Hypercholesterolemia, or high levels of cholesterol in the blood, is a significant risk factor for cardiovascular diseases, and is often caused by genetic mutations in *PCSK9* or *LDLR* [123]. PCSK9 promotes the degradation of LDLR, while LDLR removes LDL from the bloodstream. Two similar studies presented the use of LNPs to deliver an ABE mRNA and a sgRNA to knockout the expression of *Pcsk9* in mice and cynomolgus monkeys in vivo via editing of a canonical splice site [123,124]. Musunuru et al. used ABE8.8 and obtained 66% editing in monkey liver (Table 4), a 90% reduction in PCSK9 protein, and a 60% decrease in LDL cholesterol levels in the blood [123]. Rothgangl et al. used ABEmax and achieved 34% editing in monkeys (Table 4), with a 32% reduction in PCSK9, and a 14% reduction in LDL [124]. Bystander and off-target DNA editing in both reports were remarkably low. Low levels of off-target RNA editing were found, which were no longer detected 17 days after injection, since the use of LNPs is transient [124]. These observations suggested that LNP-mediated delivery of ABE mRNA is accurate and efficient enough to be optimized for use in humans. These advances opened the way for the investigational gene editing therapies VERVE-101 [141] and VERVE-102 [142] in cynomolgus monkeys, demonstrating significantly decreased PCSK9 levels and serum LDL cholesterol without affecting germline tissues, and for the ongoing clinical trials NCT05398029 and NCT06164730 by Verve Therapeutics, Cambridge, MA, USA. Furthermore, the prime editing strategy split-intein v3em PE3-AAV9, with MMR-evading silent edits, was delivered via systemic injection in the mouse *Pcsk9^Q155H^* (homolog of human *PCSK9^Q152H^*) to install a G-to-C substitution that blocks autocatalytic processing of PCSK9 and reduces PCSK9 and LDL levels [50]. This protective allele for coronary artery disease was substituted with 39% editing efficiency (Table 4), low on-target indel rates, and a 27% decrease in plasma LDL, 8 weeks post-injection. No off-target editing or liver-associated toxicities were detected. These results suggest an additional clinically relevant opportunity for the treatment of hypercholesterolemia.

Remarkably, an advanced CRISPRi approach, named evolved engineered transcriptional repressor (EvoETR), was developed to obtain durable epigenetic silencing after transient delivery of epigenome editors in mice in vivo [125]. In this work, LNPs were used to deliver dCas9 or DNA-binding zinc finger proteins (ZFPs), driving the epigenome editors of mRNA, including cdDNMT3A and DNMT3L, in addition to the KRAB domain, to silence *Pcsk9* expression in mice. Blood levels of PCSK9 were reduced by 75% until day 43 with ZFPs, and by 70% with dCas9 (Table 4). Also, a 1.9-fold increase in DNA methylation was observed at the Pcsk9 promoter in the liver, with no differences in the lung, spleen, kidney, or pancreas, confirming the targeting specificity of the LNPs. Limited off-target transcriptional and epigenetic perturbations were noted. Outstandingly, Pcsk9 silencing and repressive marks persisted after forced liver regeneration, suggesting the heritability of the newly installed epigenetic state.

In a different study, LNPs were used to deliver SpCas9 nuclease mRNA and sgRNA to disrupt the expression of *Angptl3*, which inhibits lipoprotein lipase and endothelial lipase activity, in the liver of wild-type C57BL/6 mice [126]. This approach led to a 39% editing rate (Table 4), a 66% reduction in serum ANGPTL3 protein, and a significant decrease in LDL and triglyceride levels, without off-target damage.

#### 6.7.2. Transthyretin Amyloidosis

Transthyretin amyloidosis (ATTR amyloidosis) is a progressive and fatal disease characterized by the accumulation of misfolded transthyretin (TTR) protein in tissues [143]. More than 100 pathogenic mutations in *TTR*, which is mostly expressed in the liver, are linked to the hereditary form of ATTR amyloidosis. A preclinical study reported LNP-mediated in vivo delivery of SpCas9 nuclease and a chemically modified *Ttr*-specific sgRNA in the mouse and rat models *Ttr* that resulted in 97% knockdown of serum TTR protein levels [143]. The authors also reported 70% editing in rat liver (Table 4) and 90% reduction in TTR serum levels. Low levels of editing were observed in the spleen and kidney. This opened the way for clinical trials of NTLA-2001 (NCT04601051 and NCT05697861) by Intellia Therapeutics, Cambridge, MA, USA, which showed no evidence of off-target editing in primary human hepatocytes using computational modeling, biochemical cell-free assays, and in vitro cellular assays [127].

#### 6.7.3. Tyrosinemia

Tyrosinemia is a rare genetic disorder characterized by the deficiency of enzymes involved in the breakdown of the amino acid tyrosine [128]. In the mouse model *Fah^mut^*^/*mut*^, different studies have used hydrodynamic injection of plasmids to deliver ABE6.3 with 10% correction efficiency of the mutation (Table 4), 2% bystander edits, and no off-target edits [128], or PE2 with 7% correction (Table 4) and no detectable indels, bystander edits, or off-target edits [91]. Moreover, a split PE (sPE), in which Cas9n remains untethered from RT, delivered with dual-AAV8 vectors, corrected the disease-causing mutation in *Fah*-mutant mice with a modest 1.3% editing efficiency (Table 4) [129]. In these studies, off-target edits were difficult to evaluate with confidence due to low editing efficiencies. Alternatively, higher editing levels were achieved ex vivo via plasmid electroporation to correct a mutation in *Fah* in chemically derived hepatic progenitors (CdHs) derived from, and transplanted into, the hereditary tyrosinemia type 1 (HT1) mouse model [130]. In this study, ABEmax and ABE8e reached 2.4% and 9.2% editing efficiencies (Table 4), with 29% and 11% bystander edits, respectively, without off-target damage, while PE3b reached 2.3% editing efficiency without any bystander edits (Table 4). In a different report, a PE-Cas9-based deletion and repair (PEDAR) method using two pegRNAs was developed and delivered by plasmid hydrodynamic injection into the *Fah*Δ*^Exon5^* mouse model to delete a 1.3 kb sequence, and simultaneously insert a 19 bp fragment to repair exon 5 [131]. These authors obtained only 0.76% editing efficiency (Table 4), but the growth advantage of the few corrected cells was sufficient to repopulate the liver after 40 days. The on-target indel rates caused by each pegRNA were 9.6% and 0.14%.

#### 6.7.4. Alpha-1 Antitrypsin Deficiency

Alpha-1 antitrypsin deficiency (AATD), caused by mutations in the serine protease inhibitor *SERPINA1*, is an inherited disorder that affects the production of alpha-1 antitrypsin (AAT) in the liver, which is essential for protecting the lungs from damage caused by enzymes released during inflammation [132]. In a study using Cas9 nuclease, a sgRNA and a HDR repair template was co-delivered by intraperitoneal injection of AAV8 and AAV9 in the PiZ transgenic mouse model, carrying the mutation E342K in *SERPINA1*, and a result of 2% precise correction (Table 4) and 22% on-target indels was obtained, with partial restoration of AAT in the mice serum, and without detected off-target damage [132]. In another report, Cas9 nuclease and a sgRNA, delivered systemically using AAV8 to knockout *hSERPIN1A* in the *PiZ* transgenic mouse model, led to a 98% reduction in mutant AAT expression in hepatocytes (Table 4), while using an additional AAV to provide a HDR donor template to correct the mutation led to similar results [133]. Afterwards, a group used CBE to install the compensatory mutation M374I, and in parallel ABE to correct the pathogenic mutation E342K, both delivered using LNPs and mRNA in the *PiZ* mouse model [134]. The authors noted 27% editing for CBE after 32 weeks (Table 4), 36% for ABE (Table 4), as well as increased serum AAT, improved liver histology, but also around 2% bystander mutations. No off-target edits were found in a corresponding iPSC line. Furthermore, hydrodynamic injection of plasmids was used to deliver PE2 and PE* (with an optimized nuclear localization sequence) to correct the mutation E342K in *SERPIN1A* in the *PiZ* transgenic mouse model, with 10–15% editing efficiency (Table 4) [135]. These authors also employed a split-intein AAV8-PE2 system to correct E342K with 3% editing efficiency at 10 weeks (Table 4). Off-target edits were not evaluated due to modest editing rates.

#### 6.7.5. Phenylketonuria

Phenylketonuria (PKU) is a genetic disorder characterized by the inability of the body to properly metabolize phenylalanine, caused by mutations in *PAH* [136]. An adenoviral (AdV) approach was designed to deliver a PE lacking the RNaseH domain (PE2^ΔRnH^) and correct a disease-causing mutation in the mouse model *Pah^enu2^* [136]. The authors reported an editing efficiency of 11% in vivo (Table 4), a reduction in blood phenylalanine (L-Phe), and no detectable off-target mutations or prolonged liver inflammation. However, adenovirus immunogenicity and toxicity may be problematic in terms of clinical use.

#### 6.7.6. Primary Hyperoxaluria Type 1

Primary hyperoxaluria type 1 (PH1) is a genetic disorder caused by mutations in *AGXT*, encoding alanine-glyoxylate aminotransferase (AGT), that lead to accumulation of oxalate from the liver and the formation of kidney stones and associated damage [137]. Since the disruption of *HAO1* through encoding the glycolate oxidase (GO) enzyme that synthesizes glyoxylate has proven efficient and safe in severe PH1 patients, a team has developed a way to knockout *HAO1* by systemic delivery of AAV8-SaCas9 nuclease and a sgRNA in hepatocytes of the mouse model *Agxt1*^−/−^ [137]. This resulted in >80% editing efficiency (Table 4), long-term reduction in hepatic GO and urine oxalate levels, and prevention of nephrocalcinosis, without any toxic effects or off-target damage. Another group have used the same mouse model and two AAV8 vectors delivering SaCas9 nuclease and two different sgRNAs targeting the same exon with 55% efficiency of precise deletion (Table 4), along with decreased *Hao1* mRNA and GO protein levels [138]. However, off-target indels were not systematically evaluated. In a different approach to minimizing off-target effects, a paired-D10ASaCas9n strategy was delivered with the same two sgRNAs by two AAV8 to disrupt *Hao1* in the same mouse model [139]. The authors reported 57% editing efficiency in *Hao1* (Table 4) and a lower AAV integration rate compared to Cas9 nuclease, resulting in reduced GO protein expression and decreased oxalate accumulation in PH1 mice. They did not detect any off-target modifications or DSB-mediated chromosomal translocations.

### 6.8. Immune Response Disorders

Hereditary angioedema (HAE) is a genetic disorder characterized by recurrent episodes of localized swelling (edema) in various body parts, including the skin, gastrointestinal tract, and respiratory system [140]. NTLA-2002 is an investigational Cas9 nuclease-based therapy delivered by LNPs, targeting *KLKB1* for the treatment of HAE, and developed by Intellia Therapeutics (NCT05120830) (Table 4). In the *huKLKB1* mouse model, a single administration of NTLA-2002 resulted in 70% editing (Table 4), 90% reduction in total plasma kallikrein, and abrogation of captopril-induced vascular leakage. In cynomolgus monkeys, a single administration of cyno-specific LNP formulation resulted in 70% editing and >95% reduction in both total kallikrein protein and activity. In humans, NTLA-2002 reduced total plasma kallikrein protein levels by 67–95%, with an 80–97% reduction observed in the number of monthly angioedema attacks [140].

A vast number of other studies have successfully used different gene editing tools to correct disease-associated mutations in cell models, opening the way to further preclinical studies in animal models and clinical trials in humans. As an example, a PE3 strategy delivered by electroporation in cultured human myoblasts achieved an impressive 59% correction efficiency of the mutation T4709M in ryanodine receptor 1 (*RYR1)*, which contributes to motor dysfunction and muscle weakness in *RYR1*-related myopathies (Table 3) [118].

## 7. Safety Concerns, Ethical Considerations and Regulation

While therapeutic gene editing holds great promise, it also raises safety and ethical concerns. Ensuring the safety and efficacy of these treatments, addressing ethical considerations, and promoting responsible use are crucial for realizing the full potential of gene editing in medicine. Ongoing research and clinical trials are essential to furthering our understanding and unlocking the full therapeutic potential of gene editing technologies.

### 7.1. Off-Target Effects

As mentioned above, CRISPR-Cas systems can tolerate certain mismatches between the gRNA and the target sequence. This characteristic may be essential to survive the constant acquisition of new mutations in viruses. However, it may also produce unintended mutations in patients at off-target sites with partial homology. The extent of off-target damage created by CRISPR-Cas9 nuclease, BE, or PE include on- and off-target indels in DNA, bystander base edits, off-target DNA base edits, off-target RNA base edits, and on- and off-target structural variations such as translocations, inversions, large deletions, and chromosomal rearrangements. The Cas9 nuclease approach typically produces more indels and structural variations than BE and PE due to the presence of transitional DSBs. BE is more efficient with point mutations, but also prone to bystander edits. PE is more versatile with all types of small mutations, is exempt of off-target edits, but is slightly less efficient than BE. Interestingly, a study has indicated that PE does not induce pegRNA-independent off-target mutations in the DNA or RNA, or alterations to their telomeres, endogenous retroelements, alternative splicing events, or gene expression in human cells [144]. However, since the RT embedded in the PE system produces relatively high error rates due to the lack of proofreading activity [145], caution is advised for clinical use. Even double-nicking approaches, which are tailored to prevent off-target edits, were shown to leave some on-target chromosomal aberrations [146]. All this collateral damage presents a serious concern for patients, and may complicate the development of safe gene editing therapies.

Considerable efforts have been made to develop sensitive and reliable methods for the detection of off-target edits to improve the safety of therapeutic gene editing in the clinical setting. Off-target editing can be assessed using a large variety of orthogonal in silico, in vitro, cell-based, and in vivo methods. A comprehensive overview of the advantages, disadvantages, and complementarity of different methods for off-target detection in gene editing is available [20,147,148].

Importantly, human genetic variation is considerable and should be taken into consideration when performing off-target analysis. Several of the studies described above have used mouse models to support the feasibility of editing a human transgene and have assessed off-target damage exclusively in those mice. Nonetheless, additional cell-based assays should be performed using human cells, and ideally using the patient’s target cell type. For example, analysis using neurons, directly differentiated from patient-derived iPSCs, may help in ruling out potential off-target damage for a given neurological disorder before progressing to the clinic.

To overcome the limitations of off-target effects in therapeutic gene editing, more precise algorithms can be developed to design more specific sgRNAs and pegRNAs. New modifications in Cas proteins to alter their recognition properties can improve their fidelity. Developing targeted delivery systems that bring the gene editing components specifically to the cells or tissues of interest can reduce the risk of off-target effects in other parts of the body. Strategies for controlling the timing of gene editing can reduce off-target effects by limiting the duration of enzyme activity.

### 7.2. Continuity of Expression

Long-term expression of Cas9, for example via AAV, may trigger an adverse immune response in the patient. Since the most used Cas9 orthologs come from *Staphylococcus aureus* and *Streptococcus pyogenes*, which commonly infect humans, preexisting adaptive immune responses against Cas9 may be stimulated following in vivo gene editing therapies. Indeed, antibodies as well as T cells against both SaCas9 and SpCas9 were found in >58% of donors [149], suggesting that the immune system can possibly kill the cells that are edited by the CRISPR machinery, ruining the gene editing therapy efficacy. To avoid this, transient expression of Cas9, for example by nonviral delivery, is preferred.

Since RNA base editors need to be expressed permanently to achieve their goal, they present inherent risks of continuous off-target damage. Similarly, sustained expression of DNA-targeting BE using AAV must be carefully monitored over time to ensure safety surrounding off-target base edits.

### 7.3. Ethical Considerations and Regulatory Frameworks

As gene editing technologies advance, there is a growing emphasis on ethical considerations and regulatory frameworks. The scientific community is actively engaging in discussions to address ethical concerns, potential misuse, and the responsible development and deployment of gene editing technologies.

#### 7.3.1. Germline Editing

The low selectivity of nanoparticles in most in vivo gene editing studies published so far raises a significant concern in terms of their potential to target germ cells and pass the expected edit to next generations. Germline transmission and long-term effects on future generations must be considered carefully. This type of gene editing has been met with significant hesitance from the scientific community, ethicists, policymakers, and the public for a variety of reasons that are largely independent of the caution surrounding somatic gene editing, which targets non-reproductive cells and does not affect future generations. These reasons may include unforeseen genetic issues such as off-target effects persisting across generations, or the lack of consent from individuals who will inherit the edited genes. Many countries have legal and regulatory restrictions on germline gene editing, reflecting the reluctance and ethical concerns surrounding the practice. Improving the specificity and biocompatibility of nanoparticles to target specific cells is crucial for minimizing unintended consequences.

#### 7.3.2. Regulatory Frameworks

Regulatory agencies around the world regularly release guidelines and recommendations to provide clarity on the development, testing, and approval of gene therapies. For example, Health Canada regulates cell and gene therapy products (CGTP) as biological drugs under the Canadian Food and Drugs Act and its accompanying regulations. Carefully designed clinical trials in patients must address appropriate dosing, safety parameters, and validity of surrogate or true determinants of efficacy [150]. Off-target editing still remains a risk and must be carefully addressed during preclinical studies leading to gene editing therapies. Since this class of therapy is recent, specific regulatory guidelines for off-target evaluation are not well established yet.

## 8. Future Perspectives

As gene editing technologies become more sophisticated, the range of treatable genetic disorders is likely to expand. Innovative strategies are being explored to target a broader spectrum of mutations associated with various diseases, in a more efficient and precise fashion. Higher on-target BE activity often comes with increased rates of bystander mutations, which prevents safe gene editing applications. However, the new variant NG-ABE9e, which requires a PAM composed of the nucleotides NGN, have demonstrated a seven-fold reduction in bystander editing compared with ABE8e, while maintaining similar efficiency [151]. Moreover, improvements in CBE have recently reduced off-target base edits, which may signify better performance in future experiments [152].

PE is also benefiting from recent improvements. For example, a study has revealed Exo-PE, in which an aptamer-recruited 5′-3′ exonuclease eliminates the original 5′ flap and stimulates the de novo synthesized 3′ flap to be used as a repair template [153]. This strategy circumvents the secondary nicks and possible indels that occur with PE3 and PE5. These authors indicated that Exo-PE shows higher editing efficiency than PE2 for insertions of at least 30 base pairs. Moreover, the new PE7 includes the fusion of the small N-terminal domain of the RNA-binding exonuclease protection factor “La”, and improves prime editing efficiency over PEmax, especially via RNA delivery of synthetic pegRNAs [154]. A major advantage of PE7 is to prevent the need for epegRNAs, which are still challenging to synthesize as RNA due to their longer length. The development of new PE variants holds significant promise for improving therapeutic gene editing by increasing editing efficiency, expanding targeting range, reducing off-target effects, multiplexing editing capability, enhancing delivery systems, and reducing immunogenicity.

Of curiosity, a preprint manuscript in bioRxiv currently describes the development of click editing (CE), which involves the fusion of Cas9 with DNA-dependent DNA polymerases (DDPs) and HUH endonucleases instead of RT, to install all possible small mutations, like PE does [155]. Since DNA oligonucleotides are quickly and inexpensively synthesized, this technology may facilitate and reduce the costs associated with large screening experiments. CRISPR-based technologies will likely continue to evolve.

The integration of artificial intelligence (AI) in therapeutic gene editing represents an area of growing interest and exploration, and brings new possibilities for improving component design and outcome predictions. AI can analyze genomic data from patients to identify mutations and biomarkers associated with specific diseases, and can predict optimal gRNA sequences considering the genomic context, the edit to generate, off-target sites, and possible impacts on gene function and cell phenotype, enabling personalized strategies based on genetic profiles [156]. More specifically, deep-learning-based computational models, such as DeepCas9 variants and DeepBE, have been developed to predict the efficiency and editing outcomes of various Cas9 and BE variants [157]. Furthermore, efficient PE requires size optimization of its PBS and RTT sections within the pegRNA by manually testing multiple combinations, which can be laborious and time-consuming. A group has developed an attention-based bidirectional recurrent neural network to predict pegRNA efficiencies after training on a large set of human pathogenic mutations [158]. These authors have validated their model in vitro and in vivo to predict with high accuracy the efficiency of all edit types, as well as the rates of unintended editing at the targeted loci. However, since the quantity and quality of the training data may be limited and variable in different experiments, predictive models may face uncertain reliability and interpretability. Nevertheless, the potential of AI is limitless for personalized medicine approaches in gene editing. By analyzing individual patient data, AI will certainly contribute to tailoring gene editing therapies to specific genetic backgrounds, optimizing treatment plans, and predicting potential responses or side effects.

Enhancement of RNA editing technologies, such as RNA base editing, will bring new promise for correcting genetic mutations at the RNA level, which may be relevant for diseases with DNA targets that are not easily accessible to gene editing components. Recently, a Cas13-based method named CRISPR assisted mRNA fragment trans-splicing (CRAFT) was developed to edit large 5′ and 3′ segments in different mRNAs from various mammalian cell types [159]. These authors suggest that tag fusions can be made to mRNAs using CRAFT, as well as replacing large segments of transcripts.

Future developments may include the refinement of delivery systems, improved specificity of targeting, and the incorporation of additional functionalities to enhance therapeutic outcomes. For example, extracellular vesicles (EVs) are small membrane-bound vesicles secreted by different cell types into the extracellular environment, and which are thought to be important for intercellular communication. While perhaps not yet ready for clinical applications, engineered EVs have demonstrated excellent efficiency of CRISPR delivery into various cell types in vitro [160], and are showing promising results for in vivo delivery of Cas9 [161] or CasRx [162] due to their low immunogenicity, particular biocompatibility obtained from the parent cell, and low toxicity.

In vivo gene editing to treat complex and multifactorial diseases, such as neurodevelopmental and neurodegenerative disorders, is still a challenge, but progress is being made. As gene editing therapies advance, there will be increased emphasis on regulatory frameworks and ethical considerations. Determining clear guidelines for the ethical use of gene editing and addressing societal concerns are essential for the future development of these therapies. Translation from preclinical studies to clinical trials and the eventual commercialization of widely available in vivo gene editing treatments will depend on successful clinical outcomes, regulatory approval, and accessibility. The development of patient-specific gene editing therapies and personalized medicine will improve treatment efficacy and reduce adverse effects.

In conclusion, the initial observations in the 1980s of unusual, clustered DNA repeats in bacteria led to the development of a variety of advanced gene editing technologies based on CRISPR. Cas9 nucleases are widely used and efficient in generating gene knockouts; however, this advantage comes at the price of higher off-target effects. The invention of BE has enhanced the precision of gene editing while reducing off-target indels, but is limited to single nucleotide substitutions, and may induce bystander mutations as well as off-target DNA and RNA deamination. PE is perhaps the most interesting approach for therapeutic gene editing so far since it is more versatile, addressing a wider range of mutation classes and sizes. Moreover, PE limits the likelihood of unintended changes elsewhere in the genome. High editing efficiencies, sometimes >75%, are achieved in blood and ophthalmic disorders due to greater accessibility of the target cells. Conversely, in vivo editing efficiencies are lagging behind in most neurological disorders due to several challenges like the blood–brain barrier, the complex structure of brain cells, the inactive proliferation state of neurons, and potential immune responses. The first-approved gene therapies are now being replaced by next-generation constructs with improved safety profiles and enhanced effectiveness, and their clinical use will be optimized further. Patients with rare genetic disorders that were once considered incurable will have a greater opportunity to benefit from the development of gene editing therapies.

## Figures and Tables

**Table 4:** Preclinical applications and clinical trials using gene editing for premature aging, metabolic, and immune response disorders.

Disease	Target Gene	Editor	Delivery	Editing Efficiency	Significant Unintended Edits	Drug	Clinical Trials	Sponsor	Reference
Progeria	*LMNA*	ABEmax	Split-intein dual-AAV9 in mouse *LMNA*, in vivo	30–55%	15% bystander	n/a	n/a	n/a	[122]
Hypercholesterolemia	*Pcsk9*	ABE8.8	LNP in monkey, in vivo	66%	Low	VERVE-101, VERVE-102	NCT05398029, NCT06164730	Verve Therapeutics	[123]
Hypercholesterolemia	*Pcsk9*	ABEmax	LNP in monkey, in vivo	34%	Low	n/a	n/a	n/a	[124]
Hypercholesterolemia	*Pcsk9*	v3em PE3	Split-intein dual-AAV9 in mouse, in vivo	39%	Low on-target indels, no off-target	n/a	n/a	n/a	[50]
Hypercholesterolemia	*Pcsk9*	ZFP-EvoETR, dCas9-EvoETR	LNP in mouse, in vivo	75% reduction	Limited off-target repression	n/a	n/a	n/a	[125]
Hypercholesterolemia	*Angptl3*	SpCas9 nuclease	LNP in mouse C57BL/6, in vivo	39%	None	n/a	n/a	n/a	[126]
Transthyretin amyloidosis	*Ttr*	SpCas9 nuclease	LNP in mouse and rat *Ttr*, in vivo	70%	None	NTLA-2001	NCT04601051, NCT05697861	Intellia Therapeutics	[127]
Tyrosinemia	*Fah*	ABE6.3	Hydrodynamic injection in mouse *Fah^mut^*^/*mut*^, in vivo	10%	2% bystander, no off-target	n/a	n/a	n/a	[128]
Tyrosinemia	*Fah*	PE2	Hydrodynamic injection in mouse *Fah^mut^*^/*mut*^, in vivo	7%	None	n/a	n/a	n/a	[91]
Tyrosinemia	*Fah*	split PE (sPE)	Dual-AAV8 in mouse *Fah^mut^*^/*mut*^, in vivo	1.3%	n/a	n/a	n/a	n/a	[129]
Tyrosinemia	*Fah*	ABEmax and ABE8e	Electroporation in mouse HT1 CdHs, ex vivo	2.4% and 9.2%	29% and 11% bystander, no off-target	n/a	n/a	n/a	[130]
Tyrosinemia	*Fah*	PE3b	Electroporation in mouse HT1 CdHs, ex vivo	2.3%	None	n/a	n/a	n/a	[130]
Tyrosinemia	*Fah*	PE-Cas9-based deletion and repair (PEDAR)	Hydrodynamic injection in mouse FahΔExon5, in vivo	0.8%	9.6% and 0.1% on-target indels	n/a	n/a	n/a	[131]
Alpha-1 antitrypsin deficiency (AATD)	*SERPINA1*	Cas9 nuclease (HDR)	AAV8 and AAV9 in mouse PiZ, in vivo	2%	22% on-target indels, no off-target	n/a	n/a	n/a	[132]
AATD	*SERPINA1*	Cas9 nuclease (+/−HDR)	AAV8 in mouse PiZ, in vivo	98% reduction in mutant AAT	n/a	n/a	n/a	n/a	[133]
AATD	*SERPINA1*	CBE and ABE	LNP in mouse PiZ, in vivo	27% and 36%	2% bystander, no off-target DNA, off-target RNA not looked	n/a	n/a	n/a	[134]
AATD	*AATD*	PE2 and PE*	Hydrodynamic injection in mouse PiZ, in vivo	10–15%	n/a	n/a	n/a	n/a	[135]
AATD	*AATD*	PE2	Split-intein AAV8 in mouse PiZ, in vivo	3%	n/a	n/a	n/a	n/a	[135]
Phenylketonuria (PKU)	*Pah*	PE2^ΔRnH^	AdV in mouse *Pah^enu2^*, in vivo	11%	None	n/a	n/a	n/a	[136]
Primary hyperoxaluria type 1 (PH1)	*Agxt/Hao1*	SaCas9 nuclease	AAV8 in mouse *Agxt1^−^*^/*−*^, in vivo	80%	None	n/a	n/a	n/a	[137]
PH1	*Agxt/Hao1*	SaCas9 nuclease	Dual AAV8 in mouse *Agxt1^−^*^/*−*^, in vivo	55%	n/a	n/a	n/a	n/a	[138]
PH1	*Agxt/Hao1*	D10ASaCas9n	Dual AAV8 in mouse *Agxt1^−^*^/*−*^, in vivo	57%	None	n/a	n/a	n/a	[139]
Hereditary angioedema (HAE)	*KLKB1*	Cas9 nuclease	LNP in mouse *huKLKB1* and monkey, in vivo	70%	n/a	NTLA-2002	NCT05120830	Intellia Therapeutics	[140]

PE* = nuclear localization signal sequence optimized PE2.

## Data Availability

No new data were created.
